# Recent Advances in the Application of Magnetic Nanoparticles as a Support for Homogeneous Catalysts

**DOI:** 10.3390/nano4020222

**Published:** 2014-04-02

**Authors:** Joseph Govan, Yurii K. Gun’ko

**Affiliations:** 1 School of Chemistry and CRANN, Trinity College Dublin, Dublin 2, Ireland; E-Mail: govanj@tcd.ie; 2 St. Petersburg National Research University of Information Technologies, Mechanics and Optics, St. Petersburg 197101, Russia

**Keywords:** magnetic nanoparticles, catalysis, magnetic recovery, homogeneous catalysts

## Abstract

Magnetic nanoparticles are a highly valuable substrate for the attachment of homogeneous inorganic and organic containing catalysts. This review deals with the very recent main advances in the development of various nanocatalytic systems by the immobilisation of homogeneous catalysts onto magnetic nanoparticles. We discuss magnetic core shell nanostructures (e.g., silica or polymer coated magnetic nanoparticles) as substrates for catalyst immobilisation. Then we consider magnetic nanoparticles bound to inorganic catalytic mesoporous structures as well as metal organic frameworks. Binding of catalytically active small organic molecules and polymers are also reviewed. After that we briefly deliberate on the binding of enzymes to magnetic nanocomposites and the corresponding enzymatic catalysis. Finally, we draw conclusions and present a future outlook for the further development of new catalytic systems which are immobilised onto magnetic nanoparticles.

## 1. Introduction

Catalysts play a very important role in modern science and technology as they improve reaction yields, reduce temperatures of chemical processes and promote specific enantioselectivity in asymmetric synthesis. There are two main types of catalysis, heterogeneous, where the catalyst is in the solid phase with the reaction occurring on the surface and homogeneous, where the catalyst is in the same phase as the reactants [1]. Both processes have their benefits. For example heterogeneous catalysts can be readily separated from the reaction mixture but the reaction rate is restricted due to their limited surface area [2]. Meanwhile homogeneous catalysts can react very fast and provide a good conversion rate per molecule of the catalyst, but since they are miscible in the reaction medium, it can be a painstaking process to remove them from the reaction medium [3]. The difficulty in removing homogenous catalysts from the reaction medium leads to problems in retaining the catalyst for reuse [4]. The separation and recycling of the catalyst is highly favourable since catalysts are often very expensive.

The bridge between heterogeneous and homogeneous catalysts can be achieved through the use of nanoparticles [5]. Nanoparticles of catalytic material provide the benefit of increased surface area which allows for an increased reaction rate [6]. When well dispersed, the nanoparticulate catalyst forms a stable suspension in the reaction medium allowing an elevated rate of reaction. In addition, nanoparticles can permit additional catalytic functionalities due to their unique properties, for example titania nanoparticles exhibit photooxidation on their surfaces [7] while other nanoparticles can utilize light energy as a result of their photophysical properties.

One particularly useful and important group of nanoparticles is magnetic nanoparticles (MNPs). These nanoparticles may be composed of a series of materials such as metals like cobalt and nickel, alloys like iron/platinum and metal oxides like iron oxides [8], and ferrites [9]. These nanoparticles show strong magnetic moments that are rarely retained outside of the presence of an external magnetic field. These properties are of use for a variety of potential applications such as MRI contrast agents [10,11], drug delivery [12], hyperthermal agents [13] and cell sorting [14]. There have also been reports of MNPs being used to extract selected cells from biological samples and cultures [15].

In addition to all the above, magnetic nanoparticles can serve as a highly useful catalyst support enabling immobilization and magnetic recovery of the catalyst [16]. In the absence of a magnetic field, provided there is sufficient surface stabilisation, the magnetic nanoparticles may be dispersed in the same manner as any nanoparticle. However, in the presence of a magnetic field magnetic nanoparticles can be selectively precipitated. This enables them to be readily removed from the reaction vessel by a simple magnetic separation and may enable them to be re-dispersed and re-used again.

Several types of magnetic nanostructures have developed for the use of catalysis [17,18], including the preparation of nanocomposite materials consisting of magnetic core nanoparticles which have been coated by the shell of other catalytically active nanomaterials. For example this shell coating can be photocatalytic TiO_2_ [19] as well as metal nanoparticles such as platinum [20]. These nanoparticles have shown great promise as catalysts for hydrogenation [21] and photo-oxidation [19]. In addition the magnetic core nanomaterials may also be used for inductive heating by using a high frequency magnetic field [22].

Another type of catalyst which is of interest for organic synthesis involves the use of organic molecules. These molecules show a large degree of specificity for their reactions and may allow a more successful reaction than conventional chemistry. There are two types which may potentially be used for the purposes of organic reactions, there are metal complexes which may be used to generate chemical reactions through oxidation state changes in the metal centre as well as organocatalysts which contain no metal centre and utilise conformational changes to initiate the reaction. While the metal centres may be in certain instances toxic the organocatalysts are not, but may be synthetically complex to produce, and as a result in both cases it is also desirable to retain these catalysts after use for recycling. For example, it was reported that “hypernucleophilic” 4-N,N-dimethylaminopyridine (DMAP) catalyst immobilised on magnetite nanoparticles was very active and recyclable in acyl transfer processes, Stieglitz rearrangements and hydroalkoxylation reactions at loadings between 1% and 10% and could be recovered and recycled over 30 times without any discernible loss of activity [23]. In another paper by the same group, magnetic nanoparticle-supported chiral DMAP analogue was highly active in promoting the kinetic resolution of *sec*-alcohols with synthetically useful selectivity under ambient temperature, low catalyst loading, and using acetic anhydride as the acylating agent. The use of this catalyst enabled the isolation of resolved alcohols with good to excellent enantiomeric excess. Most importantly, the interaction between the nanoparticle core and the organocatalyst unit results in a chiral heterogeneous catalyst which is recyclable to an unprecedented extent—32 times consecutive cycles—while retaining high activity and enantiomeric selectivity profiles [24].

Overall, the binding of catalysts to magnetic nanoparticles allows the retention of these materials after the end of the reaction for reuse. This will prevent the necessity of additional purification techniques to remove the catalyst from the waste stream making it a “greener” catalyst comparing to previous approaches. This area has deserved a lot of attention and there have been several good reviews on the subject of catalysts immobilised onto magnetic nanoparticles [6,16,25,26,27,28,30]. The current review considers only the most recent developments in this field during the last few years.

## 2. Immobilisation of Inorganic Catalysts on Magnetic Nanoparticles

Over the last few years significant progress was made in the development of new inorganic catalytic systems which are immobilized onto magnetic nano-carriers. Normally, to protect magnetic core and retain magnetic properties the core is coated with some non-magnetic relatively inert shell such as silica. The silica shell is very easy to be functionalized and good for binding of various catalytic species including transition metal complexes. As reported by Astruc *et al.* [31] ruthenium phosphide complexes can be easily bound between two nanoparticles through the attachment of the phosphide to the silica shell around the nanoparticles. These ruthenium complexes have shown great promise for the selective production of 1,5-disubstituted 1,2,3-triazoles and when bound to Fe_3_O_4_ nanoparticles can be reused up to five times with good yield. Pericàs *et al.* [32] have also published the functionalisation of cobalt nanoparticles with an amino alcohol that can act as a ligand for a ruthenium complex which acts as a catalyst for the asymmetric transfer hydrogenation of ketones. In their publication Wenbin *et al.* [33] reported the binding of a ruthenium complex containing diphenylethylenediamine to the surface of the magnetite nanoparticles using a phosphonic acid moiety. The resulting nanocomposites were used for the conversion of ketones to asymmetric alcohols and have been magnetically recycled up to nine times with 100% conversion and 85% conversion on the tenth run with enantiomeric excess greater than 95% in each run.

Another catalyst was reported by García-Garrido *et al.* [34] which involved a ruthenium metal centre bound to an arene as well as 1,3,5-triaza-7-phosophatricyclo[3.3.1.1.]decane also known as RAPTA. RAPTA functionalized silica coated magnetic nanoparticles were utilized for a series of microwave initiated reactions including the hydration of nitriles to nitroamides, the isomerization of allylic alcohols to ketones, and the cycloisomerization of Z-enynols to furans. In each case the reactions were conducted over several catalytic cycles with good conservation of conversion rates.

Esmaeilpour *et al.* [35] coated magnetite nanoparticles with a layer of silica and loaded a copper salen complex inside the shell, which exhibited catalysis of substituted 1- and 5-tetrazoles in high yield. In addition the same researchers [36] utilized silica coated iron oxide to bind many different metal ion species to Schiff bases to produce a series of catalysts which can be used for the synthesis of 1,1-diacetals from aldehydes. They reported yields are in excess of 90% for reactions involving the chromium (IV) catalyst.

Zolfigol *et al.* [37] developed silica coated magnetic iron oxide nanoparticles which were functionalized with palladium complexes. These nanocomposites were utilized on an aryl halide substrate for both the O-arylation of phenols and the Sonagashira reaction in aqueous media in high yield and recyclability.

Kim *et al.* [38] published the use of commercially available amine functionalized silica coated nanoparticles for the immobilisation of tridentate palladium complexes which were then used for the catalysis of dioxygenation reactions on various alkenes in microchemical set-ups. It was observed less catalyst was required in the case of the microchemical reactor than had previously been used for batch reactions.

Phan *et al.* reported the immobilization of a palladium species to the surface of cobalt ferrite nanoparticles for the use in Suzuki coupling [39] and for the Sonogashira reaction [40]. It was noted that in both cases when bound to the nanocomposite the palladium metal centre did not require the use of a phosphine ligand which is desirable due to ligand toxicity. The authors also reported that the catalysts were re-used with no significant loss of activity or leaching of palladium to the reaction medium. Other Pd based catalytic nanocomposites were produced by Gage *et al.* [41] involving the addition of an organic acid to the surface of a Fe_3_O_4_ nanoparticle and then binding to a palladium metal centre. This catalyst was used for the conversion of an alkyne to an alkene with both high yield and selectivity (both >90%).

**Figure 1:**
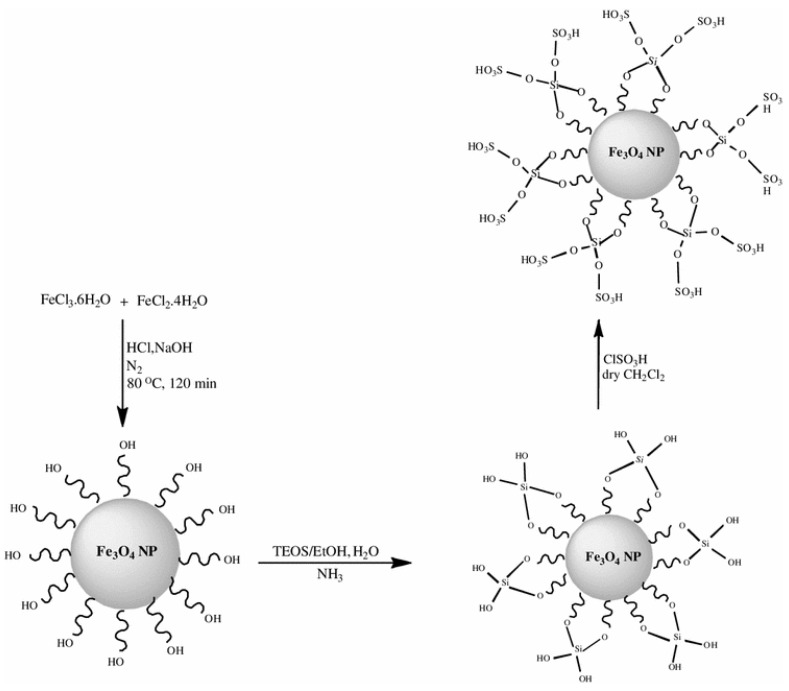
General scheme for the synthesis of silica coating and further acid functionalisation of magnetic Fe_3_O_4_ nanoparticles. Reproduced from Nazifi *et al.* [42]. Copyright 2013 with permission from Spinger.

Nazifi *et al.* [42] functionalised the surface of silica coated magnetic iron oxide nanoparticles with sulfonic acid (Figure 1) and used these nanocatalyst particles in the production of 1,8-dioxooctahydroxanthene derivatives in high yield. 

It was reported by Nakagaki *et al.* [43] that magnetite with a silica shell can act as a support for a manganese or iron containing metalloporphyrin which can act as a catalyst for oxidative reactions on cyclooctene, cyclohexene and cyclohexane. They note that the resulting nanoparticle catalysts show greater selectivity for the conversion of cyclohexane to an alcohol than free metalloporphyrin.

It was also demonstrated by Naeimi *et al.* [44] that brominated Mn porphyrins can be bound to the surface of silica coated magnetite through the use of a coordination bond between amine moieties and the manganese metal centre. The resulting nanocomposites were used for the catalysis in the epoxidation of olefins, oxidation of saturated alkanes to ketones and sulphides to sulphoxides in the presence of various oxidants. The authors analysed the recovered catalyst after seven catalytic cycles and demonstrated the retention of the porphyrin species on the magnetic support.

A silica shell was also utilised by Yoon-Sik Lee *et al.* [45] for the attachment of a nickel heterocyclic carbene catalyst to a magnetite nanoparticle. The resulting catalyst was utilised for C-S cross coupling of aryl halides with thiols in high yields. Up to three catalytic runs were reported in good yield but subsequent runs exhibited loss of Ni to the reaction medium.

However, it is not necessary for the silica shell to be inert and this might contribute to the catalysis. For example, it was demonstrated that with limited chemical modification a silica shell can also act as a mesoporous catalyst. Yeung *et al.* [46] produced mesoporous silica coated magnetic nanoparticles by a flow-through synthesis. The researchers showed that with the addition of propylamine and propyl diethylene amine these nanoparticles may be used as a catalyst in Knoevenagel condensation reactions. In another work, Abdolalian *et al.* [47] reported the development of a magnetic iron oxide nanoparticle with a mesoporous silica shell to which a molybdenum (IV) catalyst had been bound. This magnetic mesoporous catalytic system catalyses the epoxidation of olefins in the presence of hydrogen peroxide. The authors report an excellent yield in the case of cyclooctene, 1-octene, indene and 1-methylcyclohexene. The reaction was reportedly repeated up to six times with a high conversion rate. Similarly, Li *et al.* [48] bound the polyacid H_3_PW_12_O_40_ to the surface of silica coated magnetic nanoparticles providing a new catalyst system which has shown good activity in the esterification of free acids in methanol.

**Figure 2:**
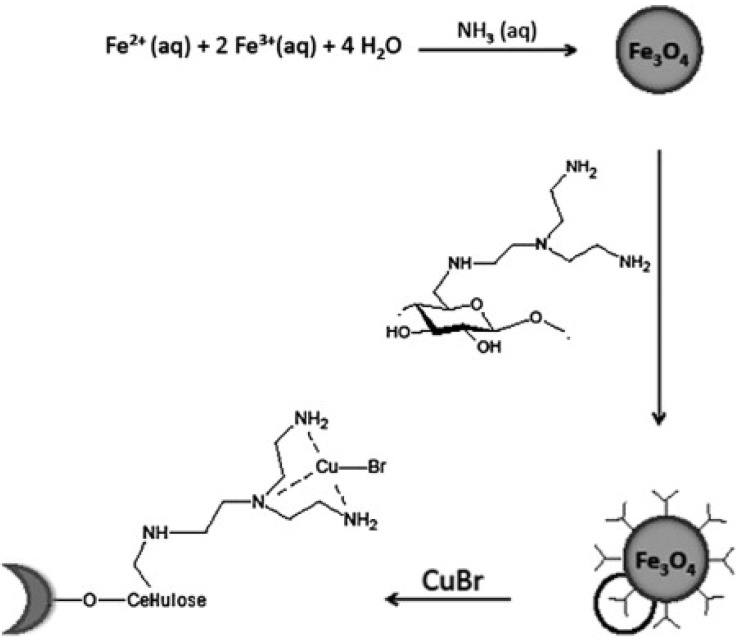
Synthesis of Fe_3_O_4_ nanoparticles bound with aminocellulose. Reproduced from Heinze *et al.* [49]. Copyright 2013 with permission of Elsevier.

The coating of nanoparticles with polymer shells was also explored in recent years. For example, an interesting catalytic system was reported by Heinze *et al.* [49]. They used aminocellulose as a coating agent for the magnetic nanoparticles which were then chemically linked to an inorganic copper bromide catalyst (Figure 2). This was used to catalyse a polymerization reaction, while the catalyst was then easily removed by magnetic separation. Wang *et al.* [50] reported the production of Fe_3_O_4_ nanoparticles which had been stabilized by poly(N-vinylimidazole). The addition of copper (II) to this polymer iron oxide nanocomposite produced a species that exhibited oxidative catalysis of 2,6-dimethylphenol in water. These species were extracted from the reaction medium with a recovery rate of over 95% and conversion rates of as high as 79.2% with supplementary catalysis.

A very interesting approach was reported by Stark *et al.* [51]. The researchers covalently bound poly-N-isopropylacrylamide to the surface of carbon coated cobalt nanoparticles. This polymer shell exhibited controllable phase transfer due to the folding of the amphiphilic polymer in response to a temperature change. The binding of a Pd-phosphine complex to the polymer shell resulted in the new “Smart catalyst”. This catalyst can be transferred from aqueous media to toluene by raising the temperature to 85 °C where it catalyses the Suzuki-Miyuara cross coupling reaction, then transferring back to aqueous media by dropping the temperature to 20 °C and collecting the catalyst by magnetic separation. The authors report the reuse of these nanocomposites up to 10 times with high conversion rates and minimal leaching of Pd into the product medium.

Commercially available polymer encapsulated magnetic beads can also be used to produce new catalytic systems. For example, Kanoh *et al.* [52] reported the coating of carboxylic-functionalised magnetic beads with a copper carboxylic acid metal-organic framework (MOF). These materials demonstrated high activity in the Henry reaction. It was reported that the reaction was repeated up to ten times while retaining good yield.

**Figure 3:**
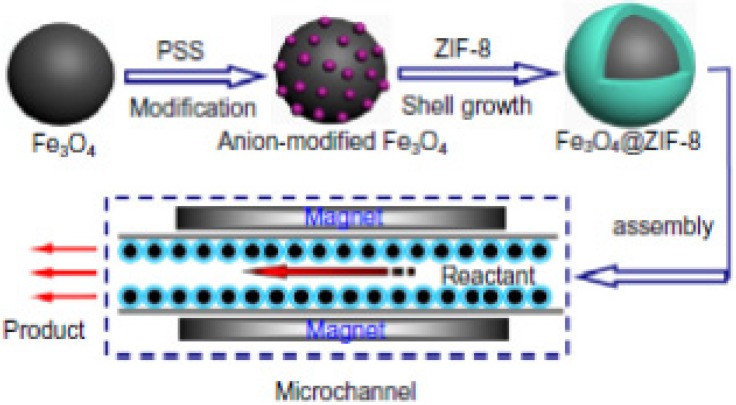
Schematic presentation of the preparation of Fe_3_O_4_@ZIF-8 core-shell microspheres and their use as catalysts in a magnetic capillary reactor. Reproduced from [53]. Copyright 2013 with permission of Elsevier.

Remarkable zeolitic framework coated magnetic nanocomposites have been used as catalysts for a flow reaction system. Yeung *et al.* [53], designed and produced these new mesoporous magnetic catalytic systems by coating of iron oxide nanoparticles with anionic polyelectrolyte layers, then adsorbing zinc cations and after that growing a zeolitic imidazolate framework (ZIF-8) by solvothermal synthesis (Figure 3). This process resulted in magnetic catalytically active microspheres (Fe_3_O_4_@ZIF-8) which were used as catalytic particles for the Knoevenagel reaction in a capillary flow reactor.

**Figure 4:**
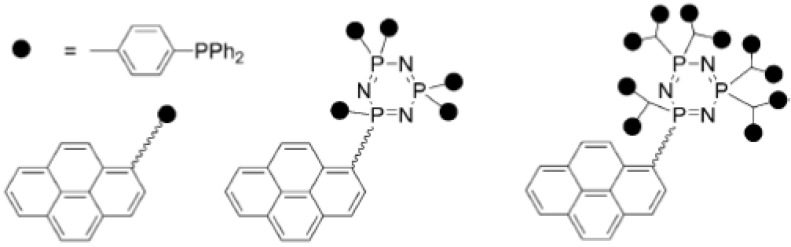
Structures of dendritic phosphine ligands. Reproduced from M. Keller *et al.* [55]. Copyright © 2013 WILEY-VCH Verlag GmbH & Co. KGaA, Weinheim.

Graphitic carbon coated magnetic nanoparticles are also very good materials for further functionalisation in catalysis applications. A highly innovative approach involving the binding of pyrene-tagged dendritic systems (Figure 4) to carbon coated magnetic cobalt nanoparticles has been developed by Reiser *et al.* [54,55]. The immobilized phosphine and dendrimer ligands on the nanoparticle surface were used to bind Pd, producing magnetically recoverable and recyclable catalysts for Suzuki coupling reactions [55]. The new catalysts were highly active in the synthesis of various biaryls, including the model synthesis of the drug Felbinac with levels of leaching of palladium within the requirements of the pharmaceutical industry (<5 ppm) [55].

The same group has reported the immobilization of hybrid palladium species on a magnetic carbon coated Co nanoparticles using ROM polymerization. This approach has resulted in generation of a magnetically recyclable palladium catalyst for Suzuki-Miyaura cross-coupling reactions [56]. Finally, very recently the same group has published two very interesting papers on the immobilization of catalytically active palladium nanoparticles on magnetic substrates [57,58]. In one of these papers palladium nanoparticles were supported on ionic liquid modified, magnetic nanobeads resulting in recyclable, high capacity catalysts for alkene hydrogenation [57]. In another work palladium nanoparticles were deposited on the surface of highly magnetic carbon-coated cobalt nanoparticles using the microwave decomposition of a Pd(0) source leading to a catalytic system with considerably higher activity in the hydrogenation of alkenes. In fact it was demonstrated that these novel magnetic nanocatalysts were superior to the industry standard Pd/charcoal in every relevant aspect including higher initial activity, more convenient separation, better recycling and less contamination of the products [58].

## 3. Immobilisation of Organic Catalysts onto Magnetic Nanostructures

As we mentioned, there is a considerable interest in the immobilization of organic catalyst molecules onto heterogeneous substrates. In this case magnetic nanoparticles offer a number of advantages as a substrate enabling the retrieval and recycling of organic catalysts which are normally impossible to remove from the reaction mixture.

**Figure 5:**
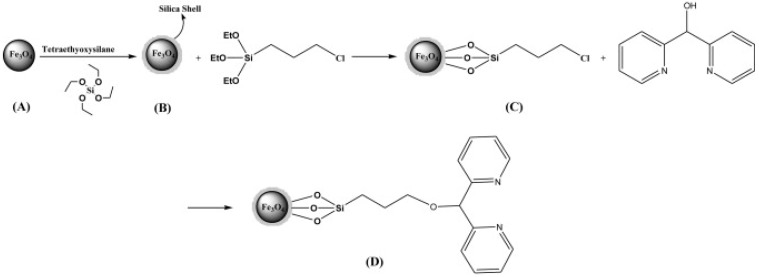
Sequence of the synthesis of Fe_3_O_4_/Dipyridine showing the original magnetic nanoparticle (**A**), the nanoparticle after attachment of a silica shell with TEOS (**B**), after the attachment of 3-chloropropyltriethoxysilane to form the organic linker (**C**), and the addition of methylene di-pyridine catalyst species (**D**). Reproduced from Nasseri *et al.* [60]. Copyright 2013 with permission of Elsevier.

As discussed above, normally organic catalysts are immobilized on the silica or polymer coated magnetic core-shell nanostructures. In this case the silica shell can both act to stabilize the magnetic core and serve as a functional substrate for the attachment of catalytic species using appropriate organosilicon precursors (linkers) [59]. For example, Nasseri *et al.* [60] bound methylene dipyridine to a silica shell on the surface of Fe_3_O_4_ nanoparticles using a triethoxysilane derivative as a linker. The nanocatalysts were applied for the synthesis of biologically active pyrazolophthalazinyl spirooxindoles in high yield (Figure 5). The authors also reported a high retention of the catalyst after successive recoveries using an external magnet.

**Figure 6:**
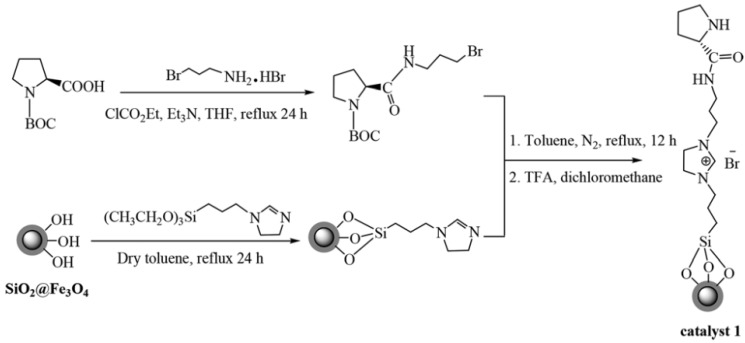
Method for attachment of proline based catalyst to silica coated magnetic nanoparticles. Reproduced from Kong *et al.* [62], with permission of the Royal Society of Chemistry.

Ponti *et al.* [61] bound an imidazolidinone to the surface of silica coated magnetite nanoparticles producing nanoparticles supported by MacMillan’s catalyst, The use of the catalyst in an asymmetric Diels-Alder reaction between cyclopentadiene with cinnamic aldehyde, resulted in the chiral products in good yields and enantiomeric excesses up to 93%. In addition the researchers demonstrated a good retention of the catalyst via magnetic separation. In another work by Yin *et al.* [62] the chiral catalyst was prepared by the addition of proline to an ionic liquid coating on silica coated magnetite nanoparticles (Figure 6). These nanocomposites have shown great promise in the catalysis of the asymmetric aldol reaction in water with high yield and enantioselectivity.

Parizi *et al.* [63] reported the attachment of a pyridine based derivative to silica coated γ-Fe_3_O_4_ nanoparticles with an organosilicon linker. This nanocomposite effectively catalyzed the production of β-phosphomalonates from diethyl phosphite and α, β-unsaturated malonates with the added benefit of catalytic action without the danger of noxious fumes from pyridine. The catalyst could also be easily recovered using a magnet and reused at least 10 times without substantial degradation in the activity.

In another work Moradi *et al.* [64] reported the production of a series of magnetite nanoparticles to which guanidine had been bound through an organosilicon linker. These catalysts have been used in conversion of aryl aldehydes and dimethyl phosphite into α-hydroxyphosphonates and with the addition of acetic anhydride, α-acetoxyphosphonates.

An organosilicon linker was also used by Karimi *et al.* [65] to bind TEMPO to magnetite which was then utilised for the oxidation of alcohols to carbonyls without the presence of metals in high yields. Up to twenty runs of the oxidation of benzyl alcohol were reported with the retention of high yields.

**Figure 7:**
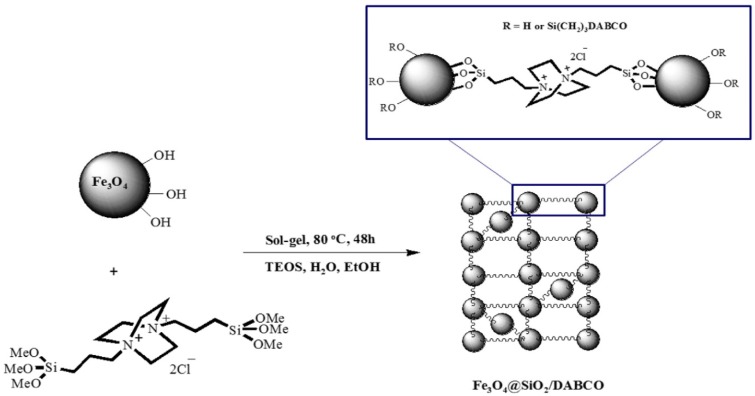
Synthesis of Fe_3_O_4_@SiO_2_/diazoniabicyclco[2,2,2]octane catalyst (DABCO) for the creation of a silica matrix. Reproduced from Kiasat *et al.* [66]. Copyright 2013 with permission of Elsevier.

Kiasat *et al.* [66] used a click-chemistry approach to generate an assembly of magnetite nanoparticles linked by a double charged diazoniabicyclco[2,2,2]octane catalyst (DABCO) within a silica matrix (Figure 7). This nanocomposite was used as a magnetically recoverable phase transfer catalyst which enabled the conversion of benzyl halides to benzyl acetates and thiocyanates in high yields without detectable side products in water.

**Figure 8:**
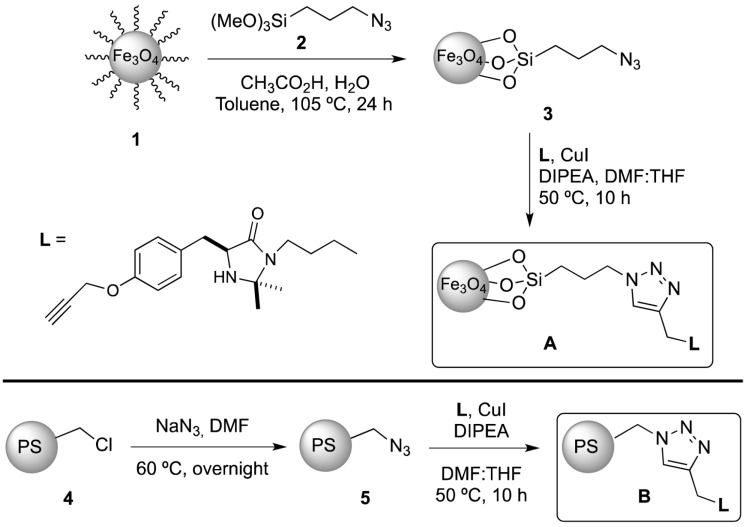
Scheme for the immobilisation of first generation Macmillan catalysts on the surface of magnetic nanoparticles and polystyrene microspheres. Reproduced from Pericàs *et al.* [67]. Copyright 2012 American Chemical Society.

Functional organic polymer coating is also good way to protect magnetic core and functionalise nanoparticles with organic catalytic species. Pericas *et al.* [67] described the use of a click chemistry approach for attaching the imidazolidin-4-one Macmillan catalyst to both polystyrene microspheres and magnetic nanoparticles (Figure 8). The catalytic ligands were linked by the copper (I) catalysed azide alkyne cycloaddition (click chemistry). These catalytic systems were then used for an asymmetric Friedel-Crafts alkylation of N-pyrroles and α-, β-, unsaturated aldehydes. While the polymer particles showed the greater level of conversion on the first run, the magnetic particles bound catalysts demonstrating a greater level of recyclability. It was also reported that the authors used a similar approach for the binding of an organocatalyst for the Micheal addition of an aldehyde to an olefin [68].

Very interesting magnetic catalytic exchange resins were prepared by Reiser *et al.* [69] using polymer coated cobalt and iron nanoparticles and microwave irradiation. The magnetic resins were employed to produce a library of ureas and thioureas using various reactions. It was demonstrated that these magnetic resins can be efficiently regenerated and reused for the next run. The same group has also reported grafting of the Jørgensen–Hayashi catalyst [(S)-α,α-diphenylprolinol trimethylsilyl ether] onto the surface with two different supports: phosphorus dendrimers and magnetic, polymer-coated cobalt/carbon nanobeads. These approaches resulted in new supported catalysts which displayed high activities and selectivities in the Michael additions of aldehydes to different nitroolefins and excellent recovery and re-use without loss of activity [70].

Another method for polymer coating of magnetic nanoparticles was reported by Seidi *et al.* [71] who used radical polymerisation to coat magnetite with a functionalised poly(ionic liquid) which was then used for the deprotection of acylals in good yields and was mentioned for possible industrial applications.

**Figure 9:**
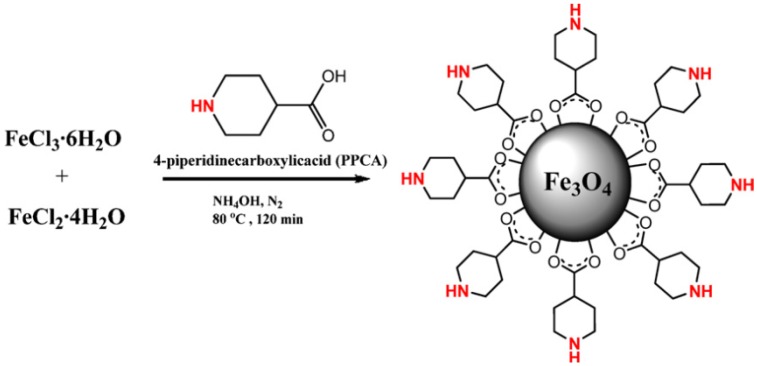
Synthesis of piperidine carboxylic acid stabilised magnetite particles. Reproduced from Toprak *et al.* [72]. Copyright 2012 with permission of Elsevier.

There are also reports on direct binding of organic catalytic species to the surface of magnetic iron oxide nanoparticles. For example, functionalised with 4-piperadinecarboxylic acid, magnetite nanoparticles were produced directly via base catalysed precipitation (Figure 9) as reported by Toprak *et al.* [72]. These nanomaterials were then used to catalyse the Knoevenagel conversion for the production of nitro-alkenes from aldehydes and nitroalkanes.

Initially, Reiser *et al.* [73] developed grafting of a stable nitroxyl radical 2,2,6,6,-tetramethylpiperidine-1-oxyl (TEMPO) to graphene-coated nanobeads with a magnetic cobalt core by using a general “click”-chemistry protocol. The new supported on Co nanoparticles TEMPO demonstrated a very high activity in the chemo-selective oxidation of primary and secondary alcohols using bleach as terminal oxidant. The catalyst could also be easily recovered by simple magnetic decantation and re-used without any further purification [73]. More recently Garrell *et al.* [74] also reported the binding of TEMPO to the surface of superparamagnetic iron oxide through a phosphate chelation and azide based click chemistry. The attachment of the catalyst to the nanoparticle surface was performed through two routes: firstly with the chelation of the phosphonate to the iron oxide surface followed by the click chemistry linkage and secondly using the click chemistry binding followed by chelation of the phosphonate at the particle surface. The resulting catalysts showed good activity in the selective oxidation of alcohols to aldehydes in both an acidic environment with Mn (II) and Cu (II) co-catalysts and in basic conditions with sodium perchlorate.

**Figure 10:**
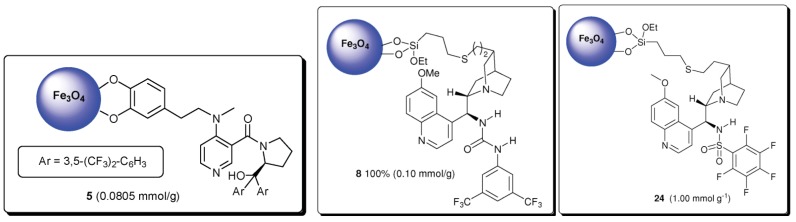
Bifunctional catalysts directly immobilised on magnetite nanoparticles. Reproduced from Gleeson *et al.* [75] with permission of the Royal Society of Chemistry.

However, as it was shown by Connon *et al.* [75], the uncoated magnetite nanoparticles cannot always be considered inert if they are functionalised by organocatalysts via direct binding to a nanoparticle surface. In this work three useful classes of chiral organocatalysts (the chiral DMAP derivative, the bifunctional (thio)urea-substituted cinchona alkaloid catalysts and the analogous sulfonamide system) were immobilised directly on the magnetite surface resulting in highly active asymmetric catalysts (Figure 10), which were tested in three different reaction classes: the kinetic resolution of *sec*-alcohols, the conjugate addition of dimethyl malonate to a nitro-olefin and the desymmetrisation of *meso-*anhydrides. Despite no physical deterioration of the heterogeneous catalysts being detected on analysis after multiple recycles, in the cases of both the conjugate addition to nitroolefins and the desymmetrisation of meso anhydrides, significant levels of background catalysis by the nanoparticles in the absence of the organocatalyst was detected. This unexpected result clearly demonstrates that magnetite nanoparticle substrates can actually participate in catalytic processes [76].

## 4. Immobilisation of Enzyme Catalysts onto Magnetic Nanoparticles

Since nanoparticles are close in size to biomolecules it is likely that they may exhibit some size related properties close to that of the molecules themselves. Enzymes are very active biomolecules, which can serve as highly specific and efficient catalysts. Many enzyme nanomimics which have been produced from nanoparticles showed specific catalytic activities [77]. Enzymes can be attached to the surface of magnetic nanoparticles or beads through the use of EDC coupling [78] and these nanocomposites can then be used for a range of reactions for the production of useful pharmaceuticals and organic materials [76,79] as well as other applications in fields such as sensing [79], for proteomic sample preparation and peptidomic analysis [80]. However, in spite of advantages enabling the retention and removal of the often expensive enzymes from the reaction medium, there are problems associated with the use of enzyme based catalytic systems with regard to their activity and selectivity and there is a need for prudence. Therefore, further innovative approaches are necessary to develop new immobilized enzymatic catalysts. 

In one recent work magnetic nanoparticles were produced via precipitation in the presence of gum Arabic by Huizhou *et al.* [81]. To these nanocomposites Lipase was bound, derived from *Candida rugosa* stabilised with surfactant. The resulting nanocomposites were then used for the multi-step synthesis of ethyl-isovalerate. Conversion rates of near 80% were reported. It was observed that across a number of catalytic cycles the bound enzyme retained greater catalytic activity than the free enzyme.

**Figure 11:**
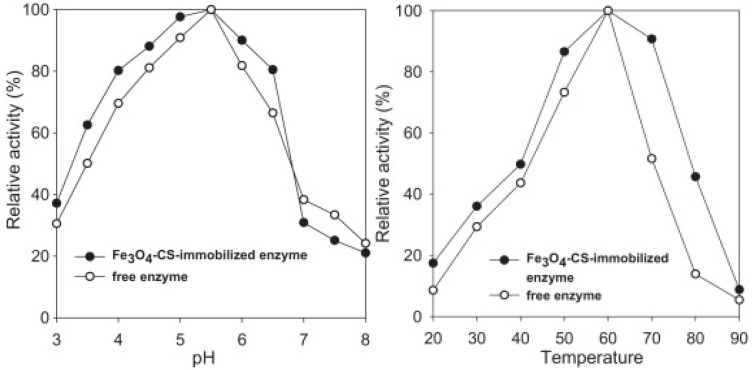
Change in enzymatic activity with changing pH (**Left**) and temperature (**Right**) to compare enzymes immobilized on nanoparticles with the free enzyme. Reproduced from Duan *et al.* [82]. Copyright 2013 with permission of Elsevier.

Chitosan stabilised magnetic nanoparticles were used by Kow-Jen Duan *et al.* [82] for the immobilisation of β-Fructanfuranosidase. This catalyst was then used for the production of fructo-oligosaccarides from sucrose. The activity of these nanoparticles was tested across various pH and temperature ranges and showed greater activity in altered conditions compared to the free enzyme (Figure 11).

Vali *et al.* [83] developed the chemical linkage of glucose oxidase to the surface of silica modified magnetic nanoparticles which were then tested for their activity over twelve reuse and regeneration cycles and were observed to have retained up to 98% activity after 45 days attached to nanoparticles. These composites were tested for both cellular and environmental assays and shown very limited toxicity.

In similar work by Kim *et al.* [84] epoxide hydrolase was bound within a framework of mesoporous silica and then used for the enantioselective hydrolysis of racemic epoxides as a method for the separation of stereoisomers. These nanocomposites retained activity over a longer time than the free enzyme. It was also noted that up to 100% conversion was reported over four cycles of use and magnetic retention.

Nanocomposites of carbon nanotubes, amyloglucosidase, and magnetic iron oxides were reported by Goh *et al.* [85] that were used for the hydrolysis of starch for use in biofuel synthesis. The bound enzymes still showed strong activity after at least a month at 4 °C in a buffer solution. A similar method was also employed by Peijun *et al.* [86] which involved the binding of a lipase from *Yarrowia lipolytica* to carbon nanotubes further functionalised with iron oxide nanoparticles. These nanocomposites were shown to be highly effective for the chiral resolution of (R,S)-1-Phenyl ethanol in heptane. They reported the resistance of the nanocomposites to sonication for up to 30 min.

## 5. Conclusions and Future Outlook

As we can see from the review above, there are some interesting new developments in catalytic systems immobilised on magnetic nanoparticles. Still there is a clear domination of the standard approach involving silica coating of magnetic iron oxide core followed by functionalisation using appropriate alkoxysilane derivatives. However, very important steps have also been made in the development of various functional polymeric coatings. In addition to mesoporous silica shells, significant progress has also been achieved in the preparation of new mesoporous zeolite-like [53] and MOF [52] coatings on magnetic nanoparticles, that enabled the combination of the advantages of porous, magnetic and molecular catalytic systems in one. We believe that this research has a great potential for the development of new multifunctional catalysts with high reactivity and selectivity.

Another interesting development is the immobilization of polydentate dendritic ligands [54,55] on magnetic nanoparticles, that enables effective removal of toxic transition metal based catalysts from important pharmaceutical products in drug synthesis. This approach should find relevant industrial applications in biopharmaceutical, food additives, fragrances and other sectors. In addition, reported by the same group new palladium nanoparticles supported on magnetic carbon-coated cobalt nanobeads are of high commercial potential and might replace the conventional and widely used palladium on charcoal reagents [58].

The report on the background catalytic activity of uncoated magnetite nanoparticles [75] serves as a serious notification for the research community working in the field of magnetically supported catalysts. However, this finding also opens up new intriguing opportunities for the development of novel hybrid inorganic/organic multifunctional nanocatalytic systems which could enable the conductance of very complex multistep catalytic transformations in one pot reactions with magnetically retrievable and re-usable catalysts.

Another important aspect is the development of new magnetically immobilised enzymatic catalysts. Despite the number of challenges and difficulties in this field, there is a great potential, particularly for biopharmaceutical applications of enzyme based catalysts.

Finally, we think that hyperthermic capabilities of magnetic substrates should be explored in the near future. This field is still very poorly developed despite the great potential opportunities to combine the catalysis with the selective local heating of reagents at the substrate. This should provide significant cost and energy savings, particularly for high temperature catalytic reactions.
